# Supplementary figures and images for: Relationship between heart rate variability parameters and inflammatory activity in patients with Crohn’s disease: a retrospective study
Source: PeerJ. 2025 Aug 18;13:e19893. doi: 10.7717/peerj.19893 (PMC12369601; doi:10.7717/peerj.19893)

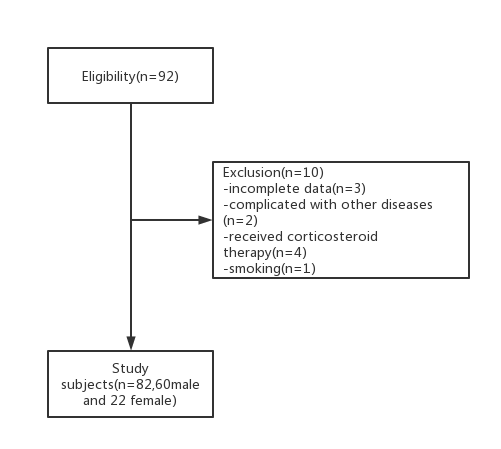

Supplement: Supplemental Information 1 [file peerj-13-19893-s001.zip › DATA/Figure 1/Figure 1.tif]

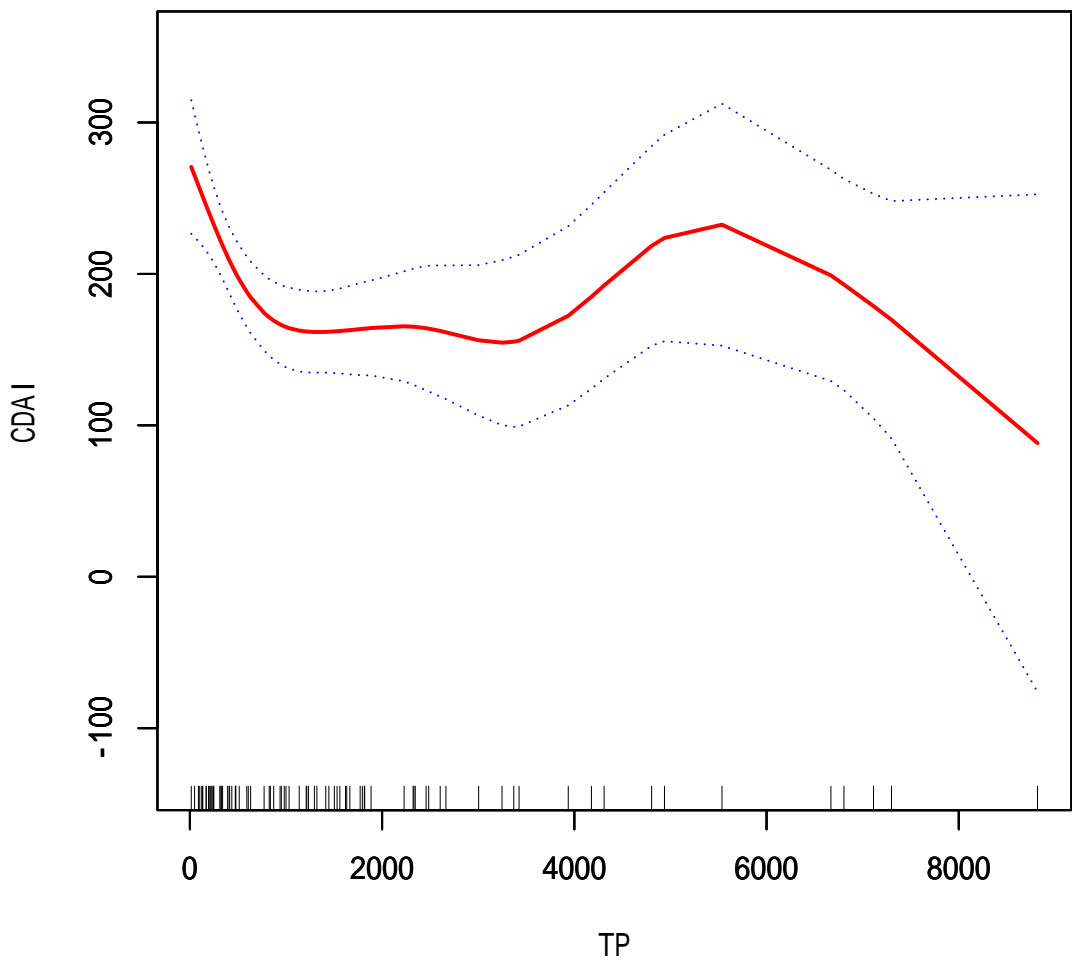

Supplement: Supplemental Information 1 [file peerj-13-19893-s001.zip › DATA/Figure 2/HRV_14_tbl_CDAI_TP_smooth1.pdf]

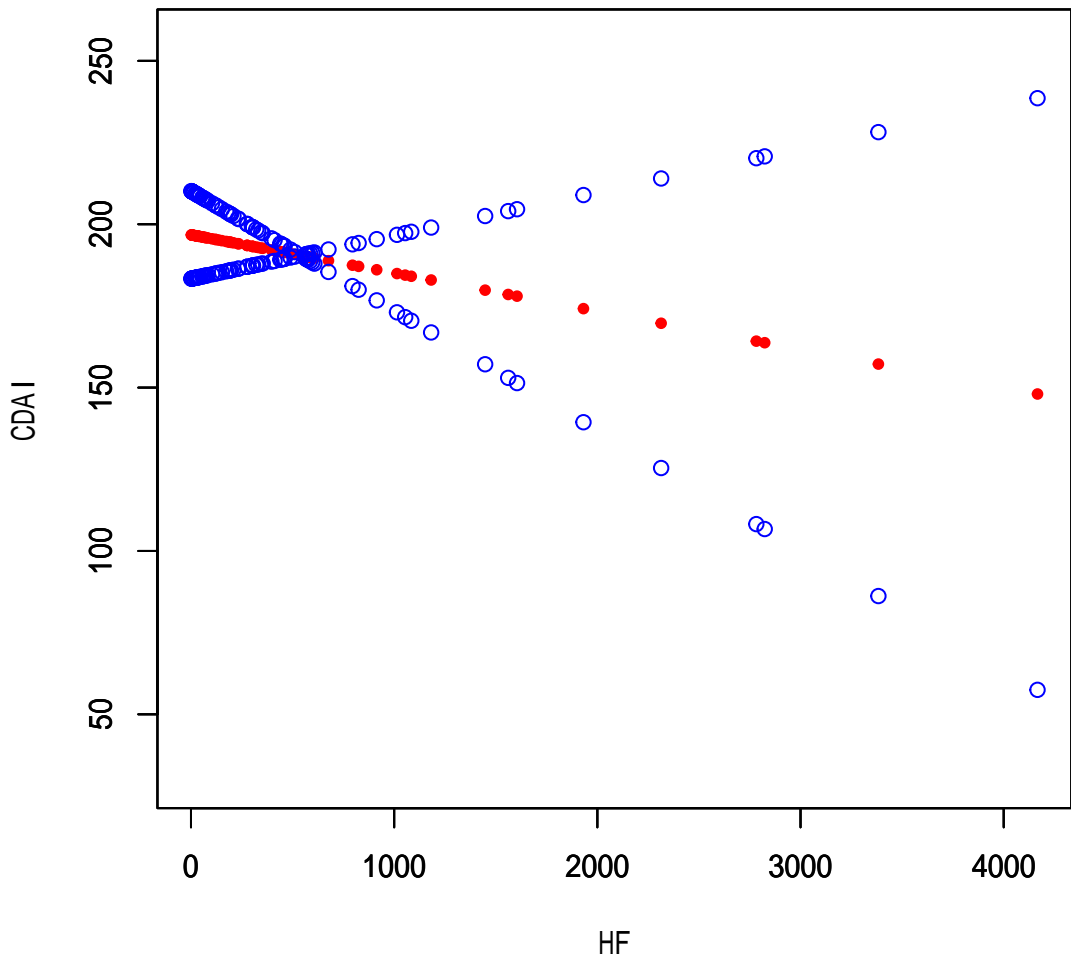

Supplement: Supplemental Information 1 [file peerj-13-19893-s001.zip › DATA/Figure 2/HRV_2_tbl_CDAI_HF_smooth.pdf]

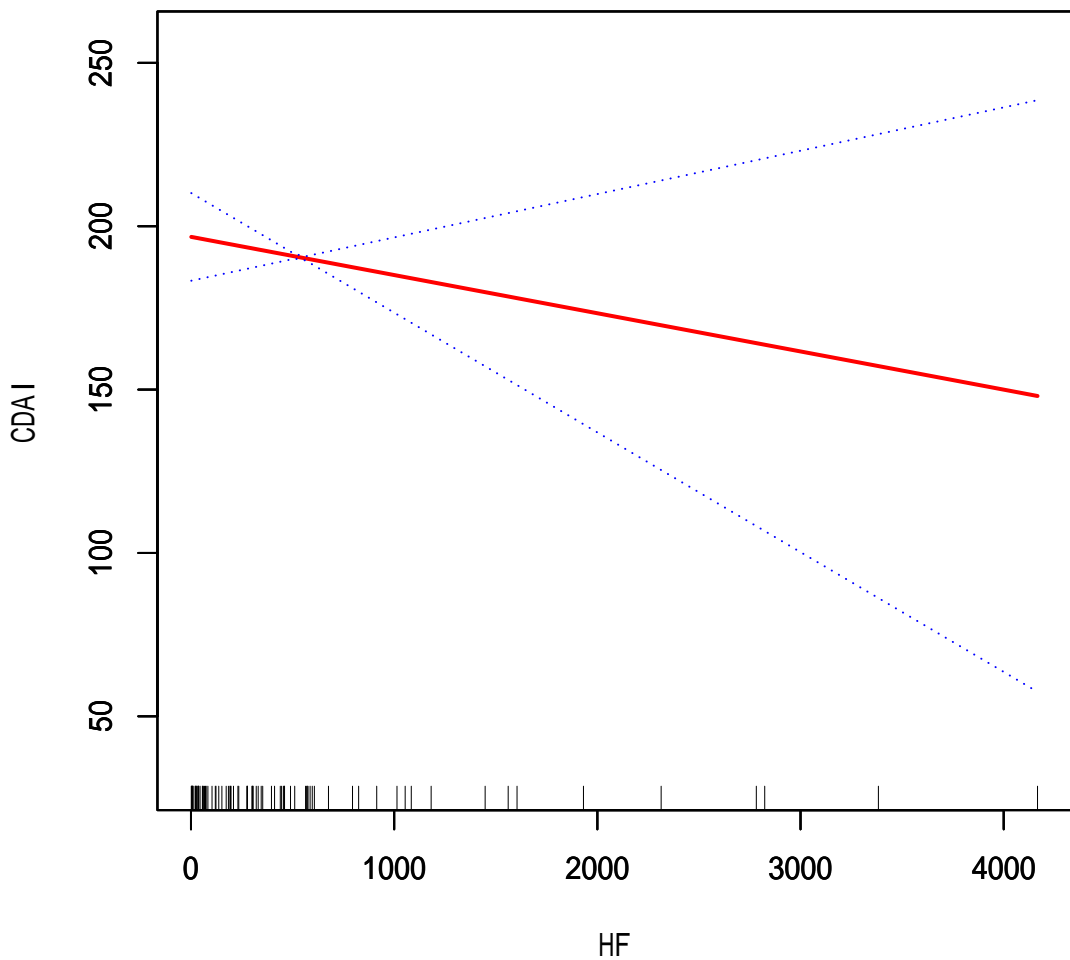

Supplement: Supplemental Information 1 [file peerj-13-19893-s001.zip › DATA/Figure 2/HRV_2_tbl_CDAI_HF_smooth1.pdf]

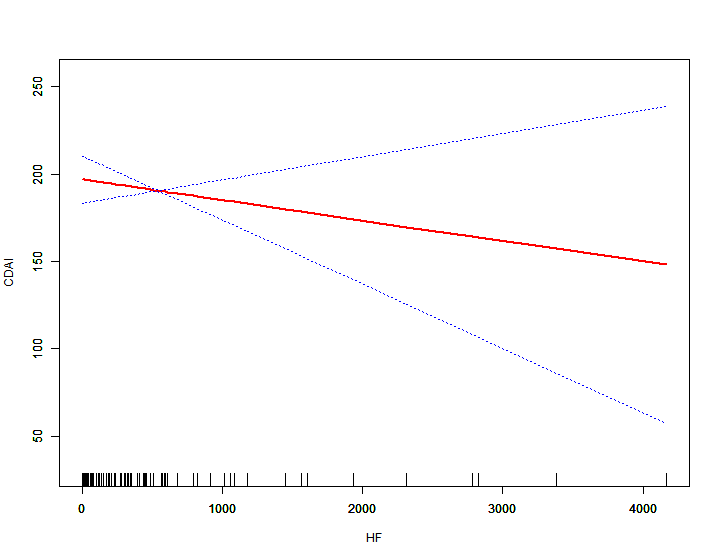

Supplement: Supplemental Information 1 [file peerj-13-19893-s001.zip › DATA/Figure 2/HRV_2_tbl_CDAI_HF_smooth1.png]

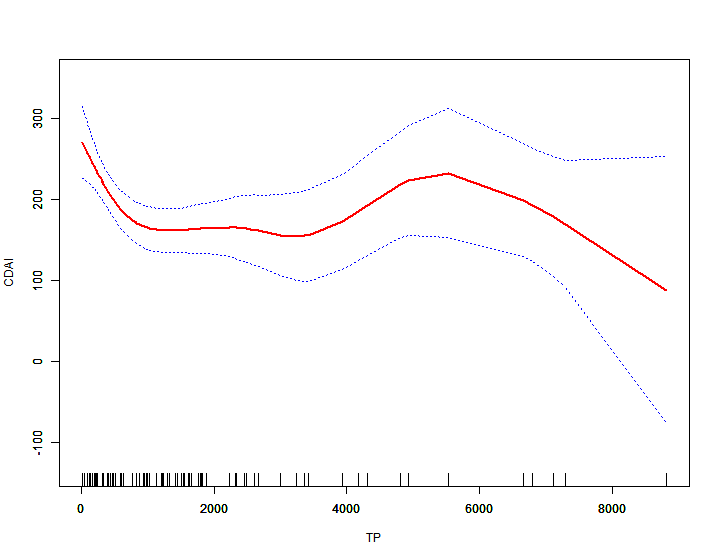

Supplement: Supplemental Information 1 [file peerj-13-19893-s001.zip › DATA/Figure 2/HRV_3_tbl_CDAI_TP_smooth1.png]

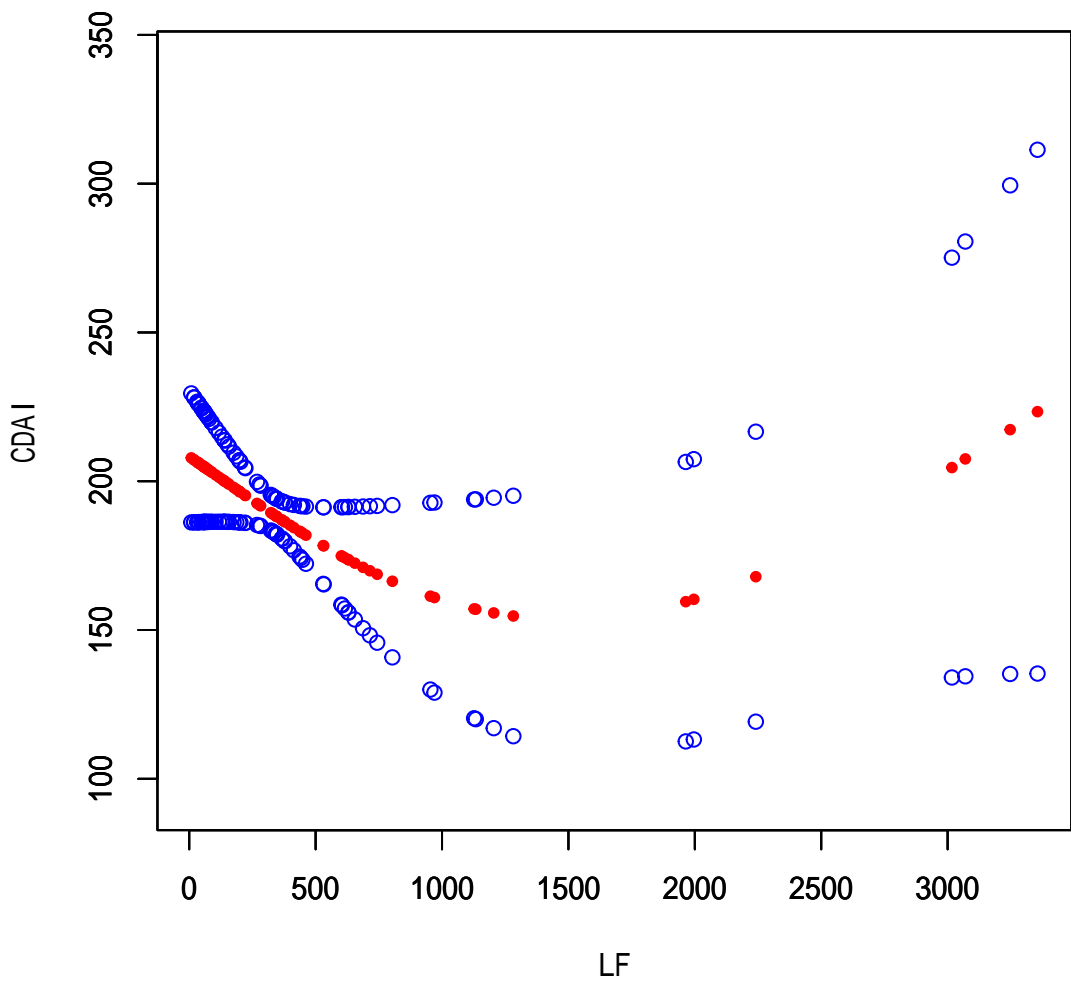

Supplement: Supplemental Information 1 [file peerj-13-19893-s001.zip › DATA/Figure 2/HRV_5_tbl_CDAI_LF_smooth.pdf]

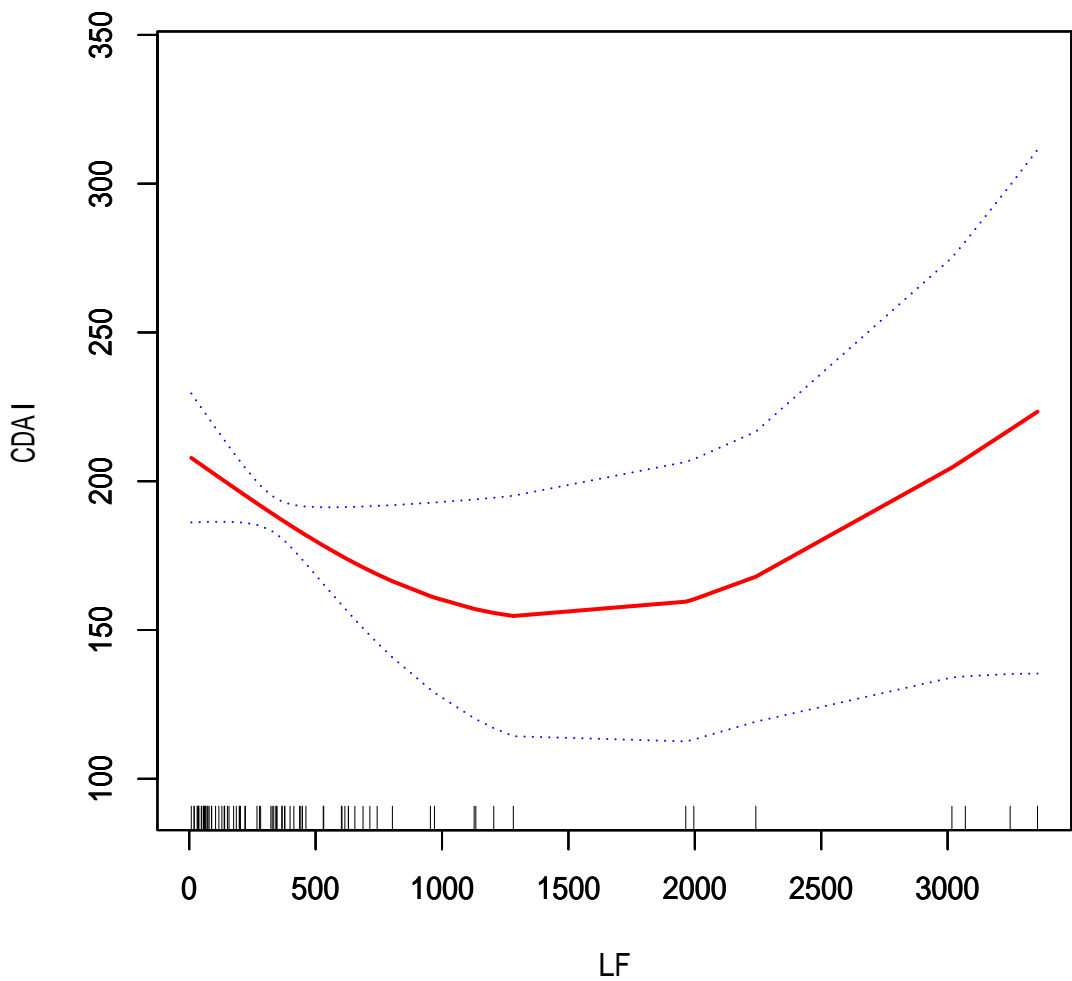

Supplement: Supplemental Information 1 [file peerj-13-19893-s001.zip › DATA/Figure 2/HRV_5_tbl_CDAI_LF_smooth1.pdf]

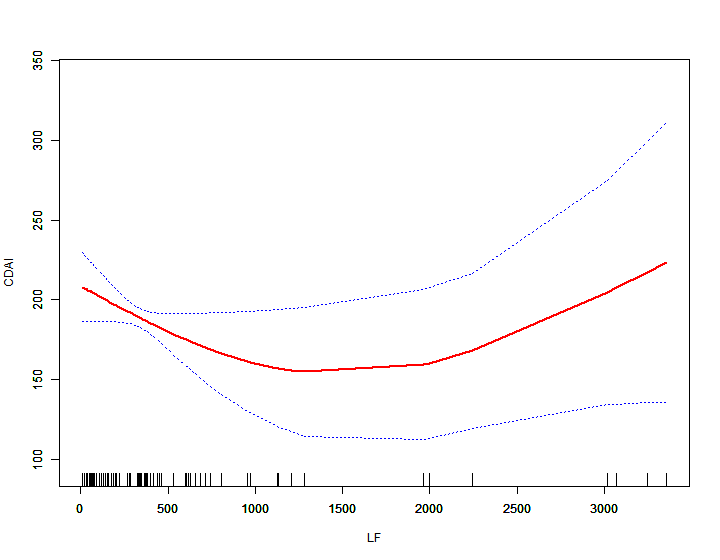

Supplement: Supplemental Information 1 [file peerj-13-19893-s001.zip › DATA/Figure 2/HRV_5_tbl_CDAI_LF_smooth1.png]

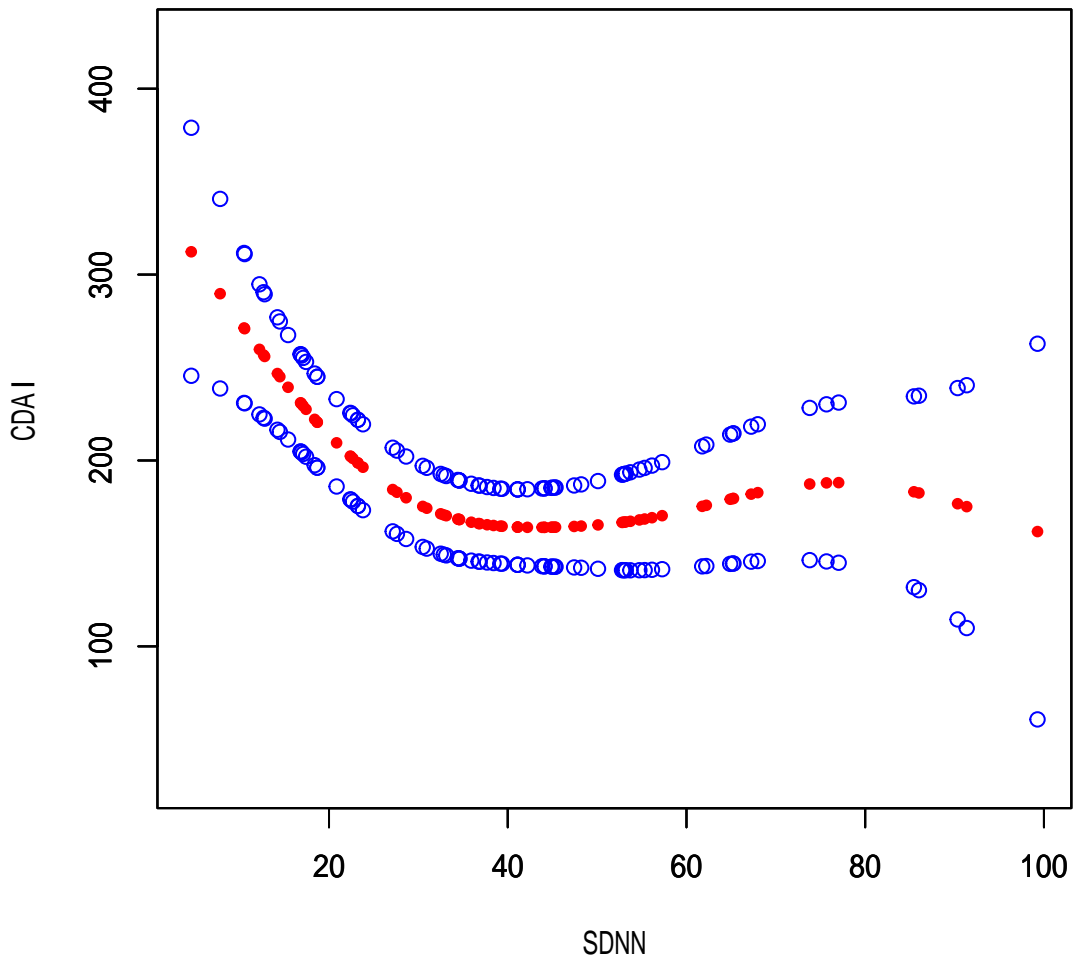

Supplement: Supplemental Information 1 [file peerj-13-19893-s001.zip › DATA/Figure 2/HRV_6_tbl_CDAI_SDNN_smooth.pdf]

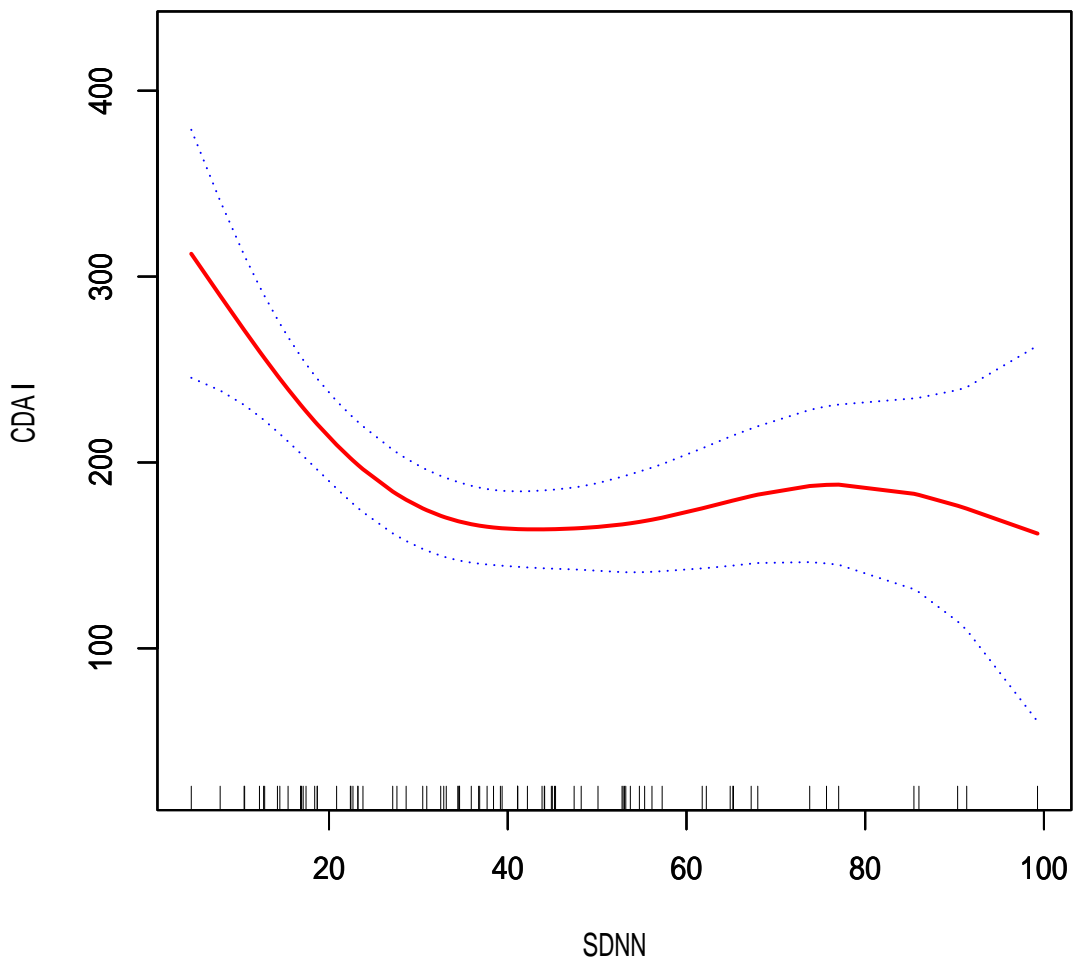

Supplement: Supplemental Information 1 [file peerj-13-19893-s001.zip › DATA/Figure 2/HRV_6_tbl_CDAI_SDNN_smooth1.pdf]

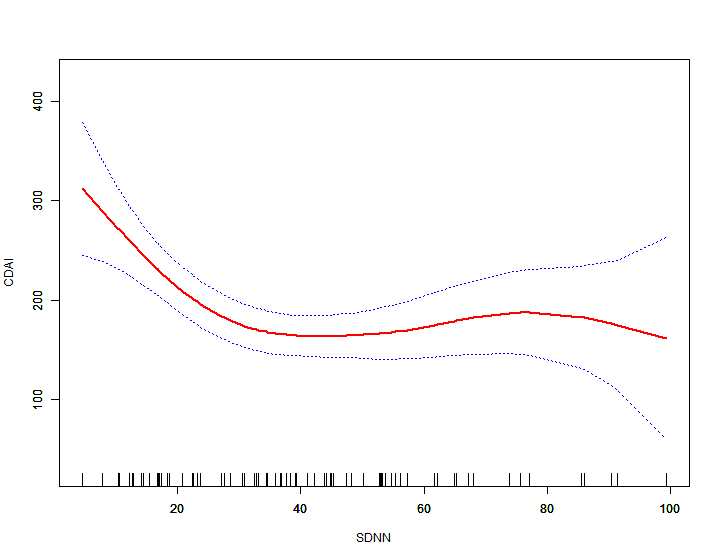

Supplement: Supplemental Information 1 [file peerj-13-19893-s001.zip › DATA/Figure 2/HRV_6_tbl_CDAI_SDNN_smooth1.png]

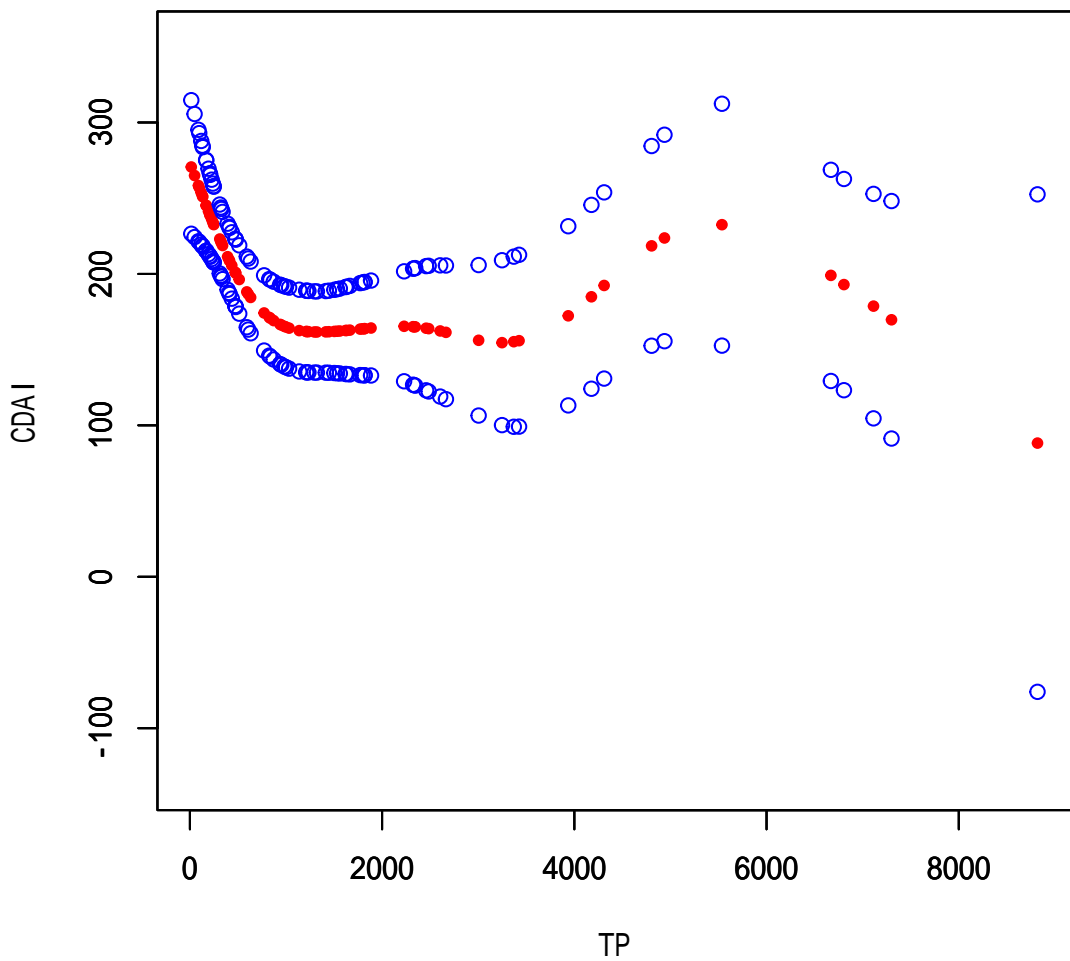

Supplement: Supplemental Information 1 [file peerj-13-19893-s001.zip › DATA/Figure 2/HRV_7_tbl_CDAI_TP_smooth.pdf]
